# Autoencoder-Based Hyperspectral Unmixing with Simultaneous Number-of-Endmembers Estimation

**DOI:** 10.3390/s25082592

**Published:** 2025-04-19

**Authors:** Atheer Abdullah Alshahrani, Ouiem Bchir, Mohamed Maher Ben Ismail

**Affiliations:** 1 Computer Science Department, Applied College, King Khalid University, Abha 61421, Saudi Arabia; 2 Department of Computer Science, College of Computer and Information Sciences, King Saud University, Riyadh 11633, Saudi Arabia; obchir@ksu.edu.sa (O.B.); mbenismail@ksu.edu.sa (M.M.B.I.)

**Keywords:** autoencoder-based unmixing, deep-learning-based unmixing, estimating the number of endmembers, hyperspectral imaging, hyperspectral unmixing

## Abstract

Hyperspectral unmixing plays a fundamental role in mining meaningful information from hyperspectral data. It promotes advancements in various scientific, environmental, and industrial applications by extracting meaningful information from hyperspectral data. However, it is still hindered by several challenges, including accurately identifying the number of endmembers in a hyperspectral image, extracting the endmembers, and estimating their abundance fractions. This research addresses these challenges by employing a convolutional-neural-network-based autoencoder that leverages both the spatial and spectral information present in the hyperspectral image. Additionally, a self-learning module utilizing a fuzzy clustering algorithm is designed to determine the number of endmembers. A novel approach is also introduced that estimates the abundances of the endmembers from the autoencoder and the clustering output. Real datasets and relevant performance metrics were used to validate and evaluate the performance of the proposed method. The results demonstrate that our approach outperforms related methods, achieving improvements of 47% in Spectral Angle Distance (SAD) and 42% in root-mean-square error (RMSE).

## 1. Introduction

Hyperspectral imaging allows the identification of unique characteristics of materials in a captured scene; thus, it is especially useful in Earth monitoring and materials detection applications related to land cover, agriculture, mineral exploration, natural disasters, and pollution [1,2]. Hyperspectral images are acquired in a process named ‘Spectroscopy’ that involves using spectroscopic sensors on aircraft or spacecraft to extract information from the Earth’s surface [2]. This process is performed by measuring the interaction of electromagnetic radiation with the materials present in a given scene. Thus, relevant information on the molecules that compose a given object/material is captured and collected [3]. A hyperspectral image can be viewed as a data cube where the spatial information is represented using two dimensions, x and y. The third dimension, λ, is the spectral wavelength dimension, which encodes hundreds of contiguous wavebands for each spatial position of a hyperspectral image. The nature of hyperspectral images imposes some limitations that require prior processing and a deeper understanding of the materials’ properties that make up a scene in order to use the data effectively [4].

The rich spectral information provided by hyperspectral imaging usually exhibits a lower spatial resolution since it depends on the sensor’s design and the distance from which the images are captured [5]. Considering that hyperspectral images are captured at high altitudes, the distance between neighboring pixels, called Ground Sampling Distance (GSD), can be very large, representing tens of meters, therefore making the objects very small [6]. This means that it is very likely that a single pixel may represent a mixture of materials. The resulting mixture is a composite spectrum constituted by pure spectra, called “endmembers”, and their corresponding contributions to the mixture are called “abundances” [7]. The nature of the spectral mixture is inherently dependent on the characteristics of the observed scene, including material composition and variability, environmental conditions, and the configuration of the sensors used. In order to identify the materials present in the scene based on their spectral properties, the overlapping spectral signatures must be unmixed [8]. The decomposition of the observed mixed pixel into a set of endmembers and the estimation of the corresponding abundances are referred to as hyperspectral unmixing. Hyperspectral unmixing is an essential, challenging step that is needed for conducting any further hyperspectral data analysis tasks [9].

Under the Linear Mixture Model (LMM), the hyperspectral mixture is assumed to be a linear combination of all materials in the scene, and the contribution of each material is directly proportional to the area it takes in the mixed pixel. Although the LMM is simple and practical in most unmixing cases, there are situations in which it may not be appropriate. Complexity arises as the electromagnetic radiation undergoes complex interactions due to the intimate mixing of materials in the scene, thus introducing nonlinear effects on the mixed spectra, which are not captured by LMMs. In addition, spectral variability, caused by factors such as illumination changes, atmospheric conditions, and intrinsic material variability, can lead to variations in the spectral signatures of the same material across the scene, further challenging the assumptions of the LMM. Such situations are usually handled using Nonlinear Mixing Models (NLMMs).

Recent efforts have addressed the limitations of LMMs by introducing nonlinear and variability-aware approaches. These methods significantly improve unmixing accuracy in complex environments. However, they often require prior knowledge of the number of endmembers or rely on supervised data, which limits their applicability.

The knowledge of the number of endmembers in a scene is essential for achieving an accurate hyperspectral unmixing. Misestimating this number negatively impacts the unmixing results: overestimating leads to representing a single material with multiple endmembers, while underestimating prevents each material from being uniquely characterized. Both scenarios result in inaccurate abundance estimation.

The process of unmixing can either be supervised or unsupervised. Supervised unmixing relies on prior knowledge about the materials’ spectra, i.e., endmembers in the targeted scene. On the other hand, unsupervised unmixing, which is called blind unmixing, aims to determine the endmembers and their abundance fractions simultaneously [10].

Although several studies have explored deep learning techniques for hyperspectral unmixing, the majority of existing approaches are supervised and rely on the assumption that the number of endmembers is known a priori—an assumption that rarely holds in practical, real-world scenarios.

This work addresses this critical gap by proposing a novel deep-learning-based blind hyperspectral unmixing framework that concurrently estimates the number of endmembers and performs the unmixing process. The key contributions of this study are as follows:

1. We propose a convolutional autoencoder-based unmixing method that operates without any prior knowledge of the number of endmembers.
2. The endmembers are extracted, and their count is self-inferred using a competitive agglomeration algorithm integrated into the learning pipeline.
3. Abundance fractions are estimated using a combination of CNN and clustering module outputs, guided by the convex geometry model.

The structure of the article is as follows: Section 2 provides a review of the existing literature on hyperspectral unmixing. The proposed methodology is described in Section 3. Section 4 outlines the experimental setup and presents the experimental results. Section 5 offers a discussion of the findings. Finally, Section 6 concludes the article and summarizes the findings.

## 2. Related Work

Unmixing approaches can be broadly classified based on their primary objectives. First, we examine hyperspectral unmixing methods that assume the number of endmembers in the scene is known a priori. Next, we discuss techniques that are specifically designed to estimate the number of endmembers from the data. Finally, we address methods that simultaneously estimate the number of endmembers and perform unmixing. The latter category presents a set of challenges, as it requires solving two complex tasks—endmember counting and hyperspectral unmixing.

### 2.1. Hyperspectral Unmixing

Hyperspectral unmixing methods can typically be grouped into two main categories: conventional linear approaches and deep-learning-based approaches. The traditional linear unmixing approaches ignore the complex multiple scattering of the electromagnetic radiation and assume that each photon reflected from the scene belongs to a single material/endmember. Hence, each pixel is a linear combination of all endmembers, weighted by its contribution to the pixel, i.e., its fractional abundance [7]. These methods include geometrical, statistical, and sparse regression methods [10]. The geometrical methods such as the N-Findr algorithm [11], the Pixel Purity Index [12], the Vertex Component Analysis (VCA) [13], and the Minimum Volume Simplex Analysis (MVSA) [14] rely on the assumption that the mixed endmember spectra are enclosed in a simplex set, i.e., a positive cone, assuming that every material/endmember in the hyperspectral image has a pure pixel.

Although simple, the geometrical methods fail when the hyperspectral image is intimately mixed, and an insufficient collection of vectors is present in the simplex facets [15]. In such cases, the statistical methods provide an alternative solution that formulates the unmixing task as a statistical inference problem. In particular, variants or extensions of the non-negative matrix factorization (NMF) [10] are adopted for blind-separation-based unmixing approaches. Alternatively, approaches that employ the Bayesian framework, such as [16,17], provide the ability to impose priors that constrain and regularize the solution space. Then, the posterior distribution of the abundances and the endmembers is computed. Other nonparametric statistical methods, as those in [18,19], exploit the Independent Component Analysis (ICA) method [20] to address the unmixing challenge. Although statistical methods are capable of unmixing intimately mixed hyperspectral images well, they suffer from a high computational cost [21].

The sparse regression methods are semi-supervised approaches where the hyperspectral data are expressed as a linear combination of known spectral signatures predefined in spectral libraries. Then, a process of finding the optimal combination of materials that best models the mixed pixel is carried out [10,15].

In these traditional linear unmixing methods, each pixel is modeled as a linear combination of fixed endmember signatures and corresponding abundance fractions. While this assumption simplifies the problem, it fails to capture the nonlinear interactions of light with complex surfaces and materials. Moreover, it assumes that each endmember is associated with a single, invariant spectral signature. This assumption rarely holds in real-world scenarios due to spectral variability [22] caused by illumination changes, atmospheric conditions, or material heterogeneity.

A variety of methods have been proposed to address these limitations. Nonlinear unmixing techniques include kernel-based approaches [23], polynomial post-nonlinear models [24], and recent deep-learning-based models [25,26] that aim to capture complex interactions in hyperspectral data. In parallel, methods such as the Endmember Bundle approach [27], Multiple Endmember Spectral Mixture Analysis (MESMA) [28], and deep generative models [29] have been developed to tackle spectral variability by allowing each endmember to be represented by a set of spectral signatures.

While these approaches have shown promise, many still rely on prior knowledge of the number of endmembers or operate under supervised settings.

The first attempt to employ deep learning to address unmixing challenges dates back to 2015 [30] when autoencoders were considered for this task. A cascade of decision models [31] consisting of a marginalized denoising autoencoder (mDA) [32] and a non-negative sparse autoencoder (NNSAE) [33] marked a successful start for using autoencoders in hyperspectral unmixing. Likewise, the work in [34] proposed a stacked set of NNSAEs to detect outliers prior to the unmixing step. Similarly, DAEN [35] is composed of two parts: stacked autoencoders (SAEs) for learning the endmembers and a variational autoencoder (VAE) for unmixing the hyperspectral image while penalizing the abundance matrix in order to fulfill the two main constraints: (i) the non-negativity constraint (ANC), and (ii) the abundances-sum-to-one constraint (ASC). Alternatively, the authors in [36] used the Spectral Information Divergence (SID) [37] as a loss function for the optimization process instead of using the Mean Squared Error (MSE) due to MSE’s sensitivity to the magnitude of the observed spectra. The latter discriminates the same endmember in a scene based on its absolute magnitude, whereas SID is not sensitive to the absolute magnitude of the endmember. Alternatively, EndNet [38], an end-to-end deep learning model that used the SAD as the loss function, showed promising results, whereas the Maximum Mean Discrepancy (MMD) was used as a regularization term in the probability metric-based autoencoder (PME) [39]. Recent works have explored advanced architectural solutions to enhance hyperspectral image unmixing. For instance, CMSDAE [40] introduced a Channel Multi-scale Processing Block (CMPB) that performs feature extraction across multiple scales at the channel level, avoiding spatial redundancy and enriching feature depth. To fuse multi-level features effectively, the authors designed the Hybrid Attention Fusion Block (HAFB), combining channel and spatial attention (via Criss-Cross attention) for adaptive, focused feature representation. Moreover, the Spectral Information Guidance (SIG) module, employing a Simplified Channel Self-Attention Mechanism (SCAM), enables long-range spectral dependency modeling.

On the other hand, CNNs were considered in the Convolutional Autoencoder for Spatial–Spectral Hyperspectral Unmixing (CNNAEU) [25] to preserve the spatial structure of the data. Similarly, the work in [41] relied on a CNN; however, it used a three-dimensional CNN instead of a two-dimensional CNN to capture spectral and spatial information simultaneously. EGU-Net [42] performs self-supervised two-stream end-member-guided unmixing. It utilizes HySime [43] to estimate the number of endmembers and uses VCA [14] as an endmember extraction method. Then, a clustering algorithm, such as K-means [44], is used to reduce the redundancy in the extracted endmembers. A similar two-stream autoencoder network was proposed in [45], employing logarithmic SAD as its loss function. This provides anomaly-based guidance that enhances the robustness of the unmixing process.

More recent work such as [46] attempted to utilize the powerful abilities of transformers to capture the global feature dependencies and preserve the spatial and spectral information in the hyperspectral image. The work in [47] combines CNNs with transformers by using the CNNs for encoding the hyperspectral image, and then a transformer with multi-head self-patch attention modules captures the contextual information in the feature dependencies of the image patches. Finally, a single convolutional layer is used to reconstruct the image. Similarly, ref. [48] combines CNNs with transformers in a comparable way by employing CNNs to extract high-level, discriminative representations, which are then converted into semantic tokens via a tokenization process. The transformer module subsequently learns the dependencies between these tokens to improve the unmixing performance. PICT [49] is a dual-stream network that utilizes transformers with the addition of prior spectral knowledge to guide the network and enhance the unmixing results. Similarly, UnDAT [50] introduces a double-ware transformer model with two modules, the score-based homogeneous-aware (SHA) module and the spectral group-aware (SGA) module. SHA creates a homogeneous map by splitting the linear feature map, while the SGA module divides the hyperspectral image into multiple spectral groups based on their spectral similarity.

### 2.2. Estimating the Number of Endmembers

Several algorithms were designed to estimate the number of endmembers in a hyperspectral image. Virtual Dimension (VD) [51], which is a class of supervised algorithms, along with the Harsanyi–Farrand–Chang (HFC) [52] algorithm, has frequently been used to find the minimum number of distinct signals in a given spectral matrix. The geometric in-degree distribution (IDD) algorithm [53] is a supervised algorithm that estimates the Intrinsic Dimension (ID) of the spectral data space in order to determine the number of endmembers. A related approach was introduced in [49], utilizing the hubness phenomenon, where the IDD is shown to be strongly influenced by the intrinsic dimensionality of the data and becomes increasingly skewed as the dimensionality rises. The HySime algorithm [15] is an unsupervised method based on the minimum error, aiming at selecting the subset of eigenvectors with the minimum root-mean-square error that best represents the signal subspace. Then, the dimensions of the obtained subspace are considered as the number of endmembers [53]. Another method based on the eigen-gap approach was proposed in [54] to estimate the intrinsic dimensionality of hyperspectral images, which corresponds to the number of endmembers. Alternatively, WGSDM [55] employs the weight-sequence geometry separation detection method, whereas [56] deploys clustering approaches to estimate the number of endmembers. On the other hand, instead of using clustering algorithms, the authors of [57] used collaborative sparsity as a promising alternative to address the overestimation of the number of endmembers in hyperspectral data typically caused by spectral variability. The work in [58] uses Iterative Error Analysis (IEA) and spectral discrimination measurements to determine the number of endmembers and remove redundancy. Recently, ref. [59] attempted to estimate the number of endmembers by detecting the intrinsic endmember spectra in the image while also eliminating the effects of the illumination-based spectral variability.

### 2.3. Simultaneous Unmixing and Number-of-Endmembers Estimation

A few attempts have been made to self-estimate the number of endmembers while performing the unmixing. These attempts can also be divided into two categories: conventional approaches and deep-learning-based approaches.

The Sparsity Promoting Iterated Constrained Endmembers (SPICE) [60] approach was introduced as an extension of the Iterated Constrained Endmembers (ICE) [61] detection algorithm that adds a sparsity-promoting term to the ICE objective function. This allows SPICE to estimate the number of endmembers and perform unmixing simultaneously. Alternatively, the Sampling Piece-wise Convex Unmixing and Endmember Extraction (S-PCUE) approach was introduced in [62]. This fully stochastic method determines the number of endmembers by estimating the number of convex regions in the hyperspectral data. On the other hand, the Mixture Analysis with Self-Estimation of the Number of Endmembers (MASENE) [63] clusters the spectral data using the Competitive Agglomeration (CA) [64] clustering algorithm. The clustering outcomes are used to derive the convex geometry unmixing model and estimate the number of endmembers. The Maximum Distance Analysis (MDA) [65] attempts to estimate the number of endmembers based on the assumption that the points and lines that are the farthest from any other point, line, or affine hull in the simplex that the hyperspectral image pixels form are endmembers. MDA also extends MVSA [14] to create MDA-MVSA, which addresses the limitations of MVSA [14], as MVSA only extracts the endmembers and requires determining the number of endmembers beforehand. However, when applied in practice, it required knowledge of the actual number of endmembers set in the parameters.

On the other hand, self-estimating the number of endmembers in a hyperspectral image and performing the unmixing task using a deep learning approach has not been sufficiently investigated in the literature. At the time of writing this research, only one deep-learning-based hyperspectral unmixing approach that self-estimates the number of endmembers was proposed in the literature. The Untied Denoising Autoencoder with Sparsity (uDAS) [21], which is a three-layer part-based denoising autoencoder proposed as an improvement to [30], addressed the issue of adopting the same regularization terms for both the encoder and decoder, arguing that the tied-weight structure hinders the unmixing process. This allows it to handle noisy hyperspectral images and reduce the redundant network weights that correspond to the extracted endmembers automatically. This yields the determination of the optimal number of endmembers in the image.

## 3. The Proposed Autoencoder-Based Hyperspectral Unmixing with Simultaneous Number-of-Endmembers Estimation

In this paper, we propose a novel hyperspectral unmixing approach that simultaneously estimates the endmembers and their respective abundances while self-learning the number of endmembers. Instead of assuming a known, fixed number of endmembers at the start of the unmixing process, we initially overestimate this number and then automatically learn the optimal value during unmixing.

More specifically, the proposed approach employs a CNN-based autoencoder to learn an effective representation of the hyperspectral image. This autoencoder is designed to leverage both spatial and spectral information to enhance unmixing accuracy. Additionally, a self-learning module based on a clustering method is integrated to automatically determine the optimal number of clusters, thereby estimating the number of endmembers in an unsupervised manner. Lastly, a new equation has been devised to estimate abundances based on the learned endmembers and their corresponding fuzzy memberships.

**Figure 1:**
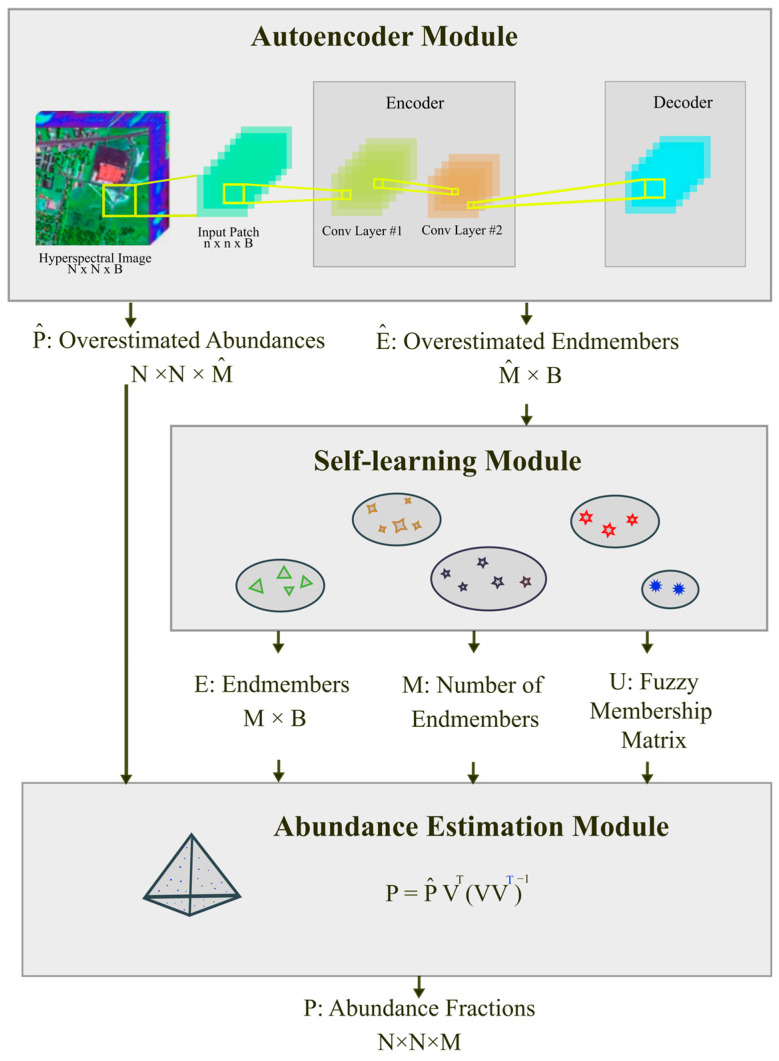
The architecture of the proposed autoencoder-based hyperspectral unmixing with simultaneous number-of-endmembers estimation.

Figure 1 illustrates the architecture of the proposed method. As shown, the autoencoder module, the self-estimation module, and the abundance estimation module constitute the three main components of the architecture.

### 3.1. Autoencoder Module

Hyperspectral images $\in R^{N\times N\times B}$ with a spatial dimension N×N, characterized by a number of bands B, are split into equal-sized patches of dimension n×n×B. Each patch, $x^{i}\in R^{n\times n\times B}$, is then conveyed to the CNN autoencoder. The choice of the CNN-based autoencoder is inspired by the work in [25], which proved to outperform other state-of-the-art approaches. Using a CNN autoencoder offers the advantage of exploiting the spatial and spectral information and combining them for a far more accurate unmixing. The encoder En is composed of two convolutional layers that aim to encode $x^{i}$ into a new feature map. Specifically, the first layer ${En}_{1}$ of the encoder with *L* number of filters can be expressed as

(1) $${En}_{1}=BN(F^{1}\left(x^{i}\right))$$

where $x^{i}$ represents the *i*th input patch, *BN* is the batch normalization transformation, and $F^{1}$ is the activation function.

Alternatively, the second layer ${En}_{2}$ of the encoder has R filters and can be defined as

(2) $${En}_{2}={\sigma \;BN(F^{2}(E}_{1}))$$

where $F^{2}$ is the activation function on the second layer and σ stands for the softmax function used to enforce the ASC. The overestimated number of endmembers corresponds to the number of filters *R* used in the second layer ${En}_{2}$. Accordingly, the result of the encoder is the overestimated abundance maps.

The overestimated endmember matrix is extracted from the kernels of the single-layer decoder with B filters. Each endmember is estimated by computing the average over the spatial indices of the kernels. This yields an endmember of size 1×B, and thus an endmember matrix of size *R × B*.

SAD is used as the loss function that measures the average of the discrepancies between the input patches $x^{i}$ and their corresponding reconstruction ${\hat{x}}^{i}$ over the K considered patches.

(3) $$mSAD=\frac{1}{K}\;\sum_{i=1}^{K}\arccos(\frac{\left\langle {\hat{x}}^{i},x^{i}\right\rangle }{{\left\|{\hat{x}}^{i}\right\|}_{2}{\left\|x^{i}\right\|}_{2}})$$

SAD is a scale-invariant metric well suited for hyperspectral unmixing tasks, as it does not penalize the natural spectral scale variabilities present in hyperspectral images. However, it is sensitive to differences in the spectral shape, which it does penalize. Algorithm 1 outlines the steps involved in the autoencoder module.
**Algorithm 1:** Autoencoder module**Input:** Hyperspectral data cube $X\in R^{N\times N\times B}$, number of filters in 1st layer *L*, number of filters in 2nd layer *R* **Output:** Overestimated endmembers, $\hat{E}\in R^{R\times B}$, their abundance, $\hat{P}\in R^{N\times N\times R}$
Split X into n×n×B patches $x^{i}$Train the CNN autoencoder using $\left\{x^{i}\right\}$ to learn $\hat{P}$Extract the encoder layer (latent space) and assign it to the overestimated abundance $\hat{P}$Extract the decoders’ weights and assign them to the overestimated endmembers $\hat{E}$

### 3.2. Self-Learning Module

The matrix $\hat{E}\in R^{R\times B}$, learned using the autoencoder module, typically contains an overestimated number of endmembers due to duplication. To address this, we propose automatically eliminating the duplicated endmembers by clustering the spectral signatures. However, since the actual number of endmembers—corresponding to the number of clusters—is unknown, we propose to simultaneously estimate the number of endmembers during the clustering process. For this purpose, we adopt the CA clustering algorithm [64]. Specifically, a fuzzy membership matrix $U\in R^{R\times R}$ is initialized to represent the degree to which each endmember belongs to each cluster, where R is the initial (overestimated) number of endmembers. The R cluster centers $\left\{E_{i}\right\}$ are then computed as follows:

(4) $$E_{i}=\frac{\sum_{i=1}^{R}U_{ij}^{m}{\hat{E}}_{j}}{\sum_{j=1}^{R}U_{ij}^{m}}$$

where $\hat{E}\in R^{R\times B}$ is the set of overestimated endmembers, $U_{ij}$ is the membership degree of endmember ${\hat{E}}_{j}$ with respect to cluster i, and m is a fuzzifier typically set to 2. Then, the fuzzy memberships are re-updated using

(5) Uij=1distEi,E^j∑q=1R1distEq,E^j+αdistEi,E^j(Ni−N¯)

where $dist\left(E_{i},{\hat{E}}_{j}\right)$ is the distance between the endmember ${\hat{E}}_{j}$ and the cluster center $E_{i}$ and α is a weighting factor used to balance the terms in the clustering objective. $N_{i}$ denotes the cardinality of cluster i, defined as the sum of the membership values for that cluster, and $\bar{N}$ stands for the average cardinality across all clusters.

After the fuzzy membership matrix is updated, the number of endmembers is adjusted by modifying the number of clusters. The CA clustering algorithm evaluates the cardinality of each cluster in every iteration and eliminates clusters with cardinality below a specified threshold. However, due to the limited number of instances (i.e., the overestimated number of endmembers), the dense data topology, and the high dimensionality of the spectral signatures, this CA-based elimination strategy proved to be ineffective. Therefore, this research investigated four clustering homogeneity measures to discard irrelevant clusters. Namely, the Fuzzy Partition Coefficient (FPC) [61], Silhouette Score (SS) [62], Calinski–Harabasz Score (CHS) [63], and Davies–Bouldin Score (DBS) [64] were used in this research. The cluster centers, the fuzzy memberships, and the number of clusters are updated iteratively until convergence. The resulting number of clusters corresponds to the learned number of endmembers *M*, and the final cluster centers represent the estimated endmembers $\left\{E_{i}\right\}$. The steps of the self-learning module are outlined in Algorithm 2.
**Algorithm 2:** Self-learning module**Input:** Overestimated endmembers $\hat{E}\in R^{R\times B}$ **Output:** Number of endmembers, M, endmembers, $E\;\in R^{M\times B}$
Initialize the fuzzy membership matrix $U\in R^{R\times R}$Repeat until convergence:   Update the cluster centers E using (4)   Update the membership matrix *U* as (5)Update the number of clusters M

### 3.3. Abundance Estimation Module

In order to estimate the abundance fractions $P\in R^{N\times N\times M}$, the convex geometry model is employed as follows:

(6) $$E_{i}=\sum_{j=1}^{R}\frac{{\left(U_{ij}\right)}^{m}}{\sum_{q=1}^{M}{\left(U_{iq}\right)}^{m}}{\hat{E}}_{j}$$

where *R* is the number of overestimated endmembers and *M* represents the learned number of endmembers. $\hat{E}\in R^{R\times B}$ is the matrix of overestimated endmembers, while *E* stands for the matrix of learned endmembers. The model in (6) can be re-formulated using the following matrixial form:

(7) $$E=V\;\hat{E}$$

where each entry, $v_{ij},$ of the matrix V is defined as

(8) $$v_{ij}=\frac{U_{ij}^{m}}{\sum_{q=1}^{N}U_{ij}^{m}}$$

According to the convex geometry model, one can write

(9) $$X=\hat{P}\;\hat{E}$$

where $\hat{P}$ is the matrix of overestimated abundances learned by the autoencoder module.

Given (7) and (9), we obtain

(10) $$PE=\hat{P}\;\hat{E}$$

Furthermore, we replace *E* using (7) and obtain

(11) $$P\;V\;\hat{E}=\hat{P}\;\hat{E}$$

This yields

(12) $$P\;V=\hat{P}$$

Lastly, we can calculate the abundance maps as follows:

(13) $$P=\hat{P}\;V^{T}{\left(VV^{T}\right)}^{-1}$$

Algorithm 3 outlines the steps of the proposed fraction estimation module.
**Algorithm 3:** Fraction estimation module**Input:** Learned endmembers $E\in R^{M\times B}$, overestimated endmembers $\hat{E}\in R^{R\times B}$, overestimated abundances $\hat{P}\in R^{N\times N\times M}$, the fuzzy membership matrix $U\in R^{M\times R}$ **Output:** Abundances, $P\in R^{N\times N\times M}$
Compute V using (8)Compute P using (13)

## 4. Experiments

Three hyperspectral datasets were considered to assess the performance of the proposed approach. Namely, the Urban dataset with its three variations, Urban4, Urban5, and Urban6 [66], the Samson dataset [67], and the Jasper Ridge dataset [68]. The wide use of these datasets by the hyperspectral unmixing community can be attributed to the availability of the ground truth endmember signatures. The characteristics of the considered datasets are summarized in Table 1.

**Figure 2:**
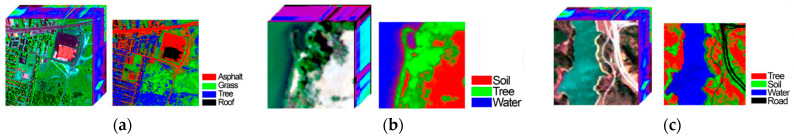
The pseudo-RGB representation of the (**a**) Urban dataset, (**b**) Samson dataset, and (**c**) Jasper dataset.

**Figure 3:**
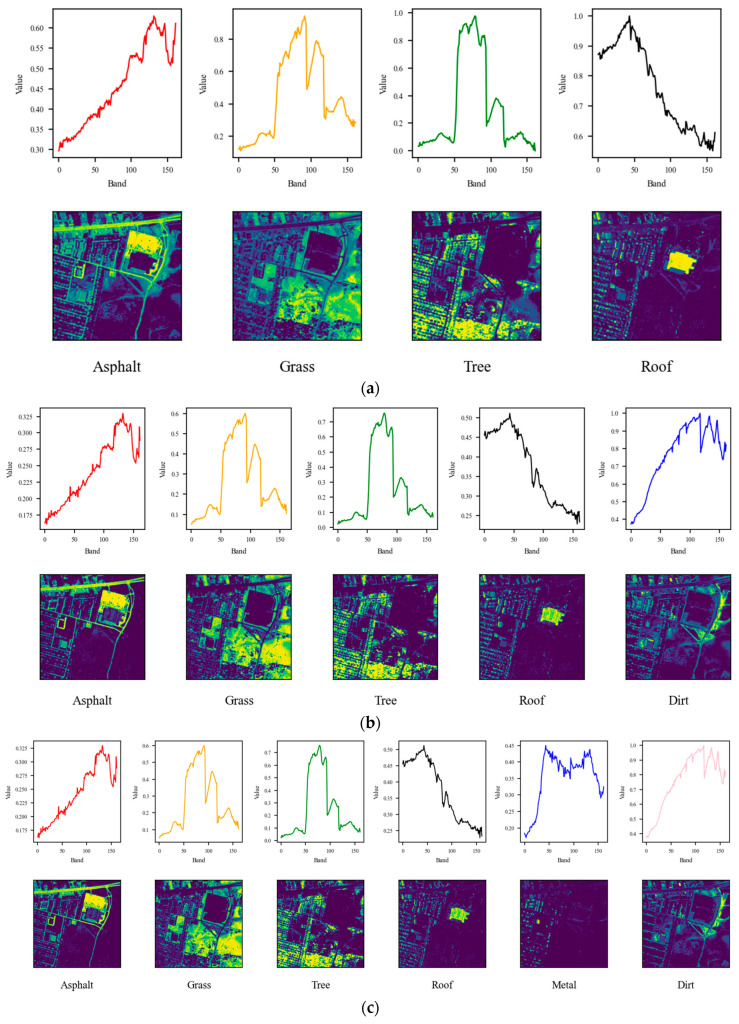
Endmembers and abundance maps of the urban dataset variations: (**a**) Urban4, (**b**) Urban5, (**c**) Urban6.

**Figure 4:**
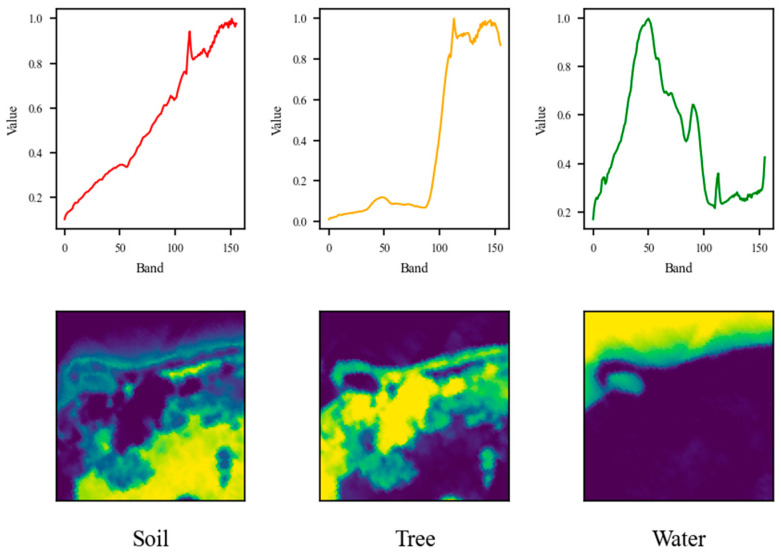
Endmembers and abundance maps of Samson dataset.

**Figure 5:**
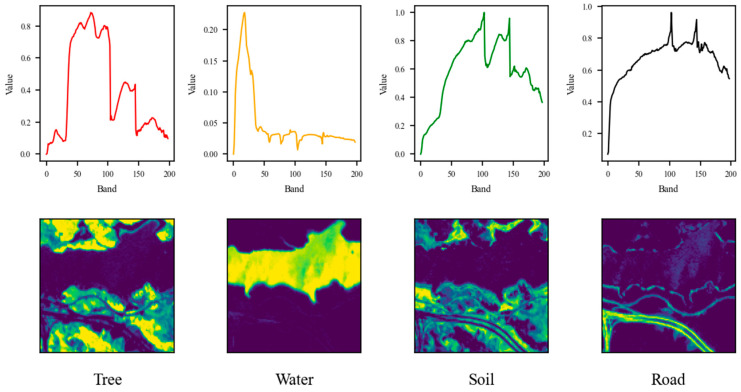
Endmembers and abundance maps of Jasper dataset.

The pseudo-RGB representations of the datasets are shown in Figure 2, while the ground truth endmembers and abundance maps are shown in Figure 3, Figure 4 and Figure 5.

First, we investigated unmixing the hyperspectral data cube using the first module (i.e., the autoencoder) with an overestimated number of endmembers. The experiments were carried out using the hyperparameters outlined in Table 2.

**Figure 6:**
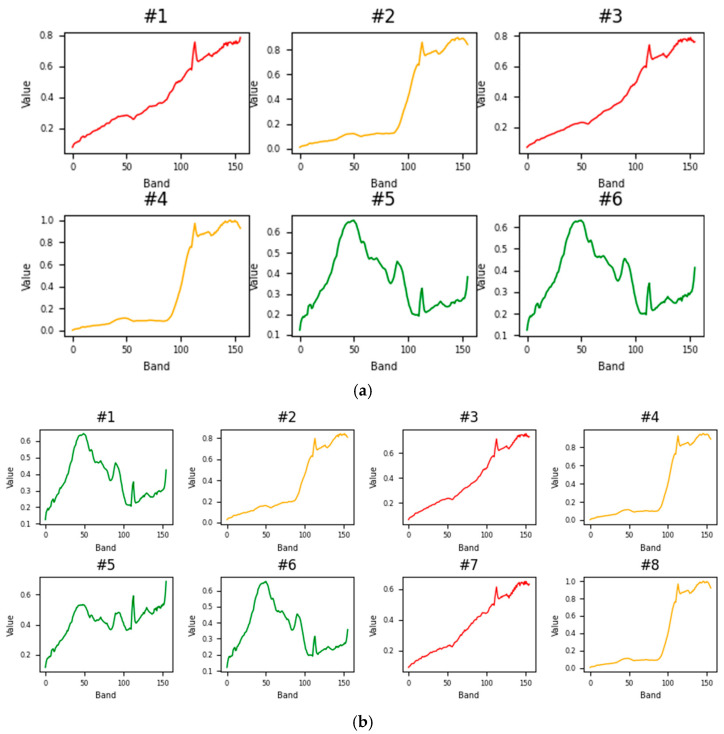

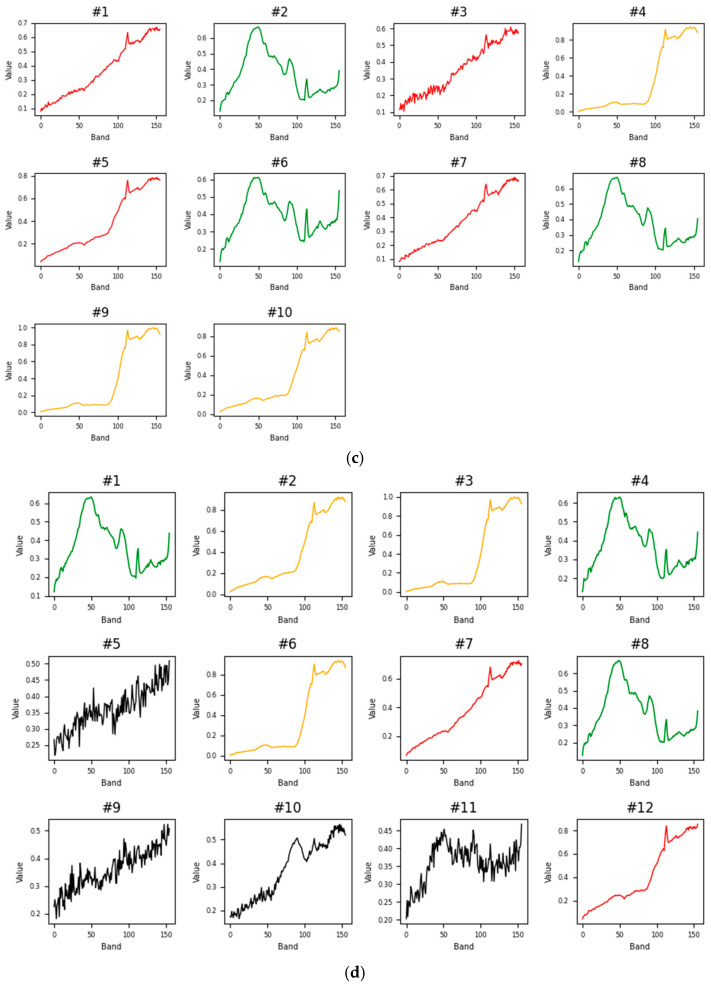

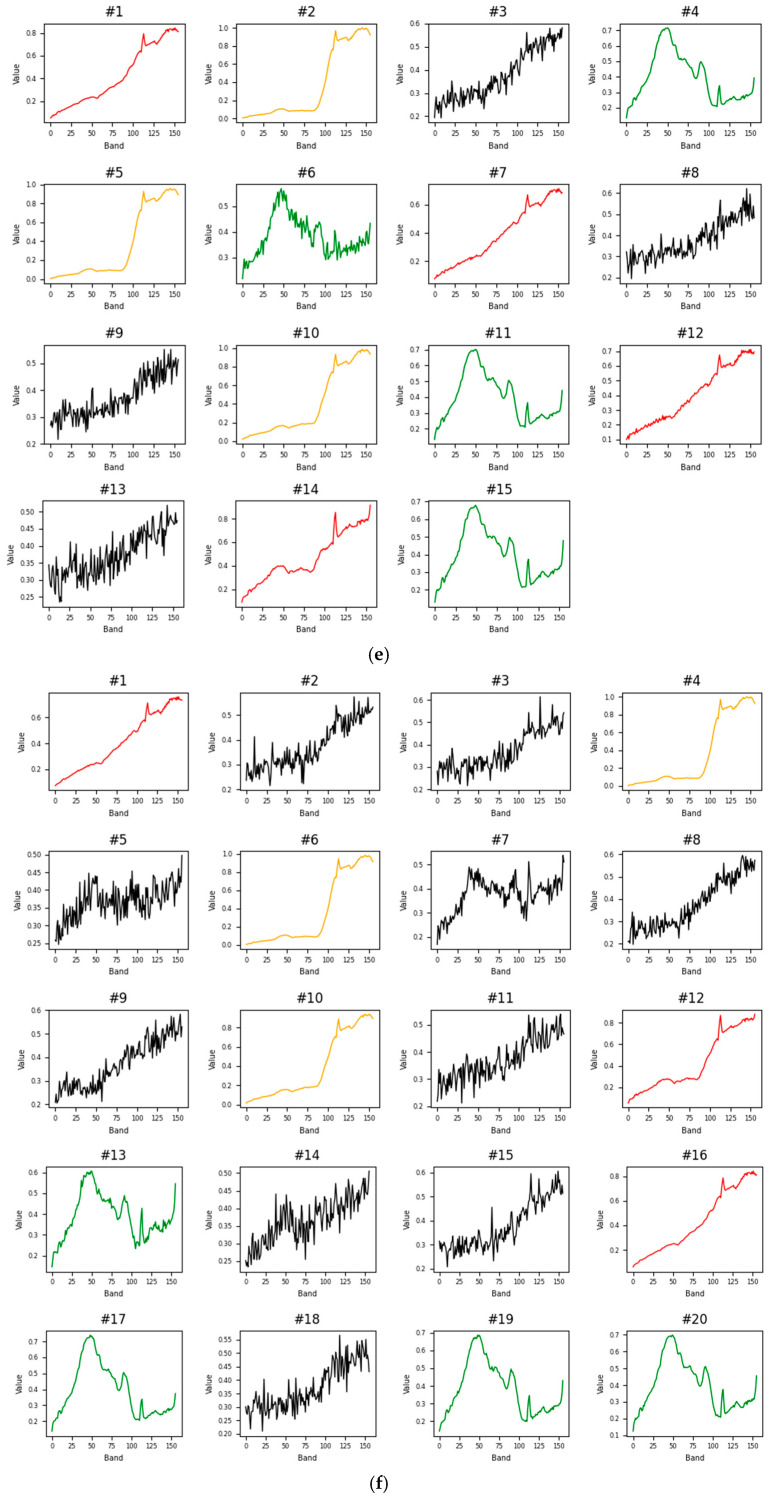
The endmembers determined for the Samson dataset given an overestimated number of endmembers set to (**a**) 6, (**b**) 8, (**c**) 10, (**d**) 12, (**e**) 15, and (**f**) 20.

Six experiments were conducted using the Samson dataset. In each experiment, one of the following values—6, 8, 10, 12, 15, and 20—was used as the initial number of endmembers. Figure 6 illustrates the resulting endmembers. As shown, the obtained endmembers are redundant, with similar endmembers represented by the same color. Additionally, when the number of endmembers is overestimated to 12, 15, or 20, additional signatures (in black) that are dissimilar to those in the ground truth data are extracted. This highlights that overestimating the number of endmembers not only leads to duplication but also produces fictitious signatures. Similar results were observed when conducting the same experiments on other datasets, where the obtained endmembers were not only duplicated but also included fictitious ones.

To quantitatively assess the effect of overestimating the number of endmembers on the considered datasets, we computed the SAD score between the learned endmembers and the ground truth endmembers. Since the number of extracted endmembers exceeds the ground truth value, all possible combinations of the obtained endmembers were evaluated. Specifically, the SAD score was computed by comparing the ground truth endmembers with each combination of the learned endmembers. Table 3, Table 4, Table 5, Table 6 and Table 7 report the minimum SAD for all combinations, as well as the average SAD and the corresponding standard deviations for the five datasets.

As observed, the average SAD increases as the number of endmembers rises, indicating that overestimating the number of endmembers leads to inaccurate unmixing results. However, when considering the minimum SAD across all combinations, the SAD decreases as the number of endmembers increases, which corresponds to more accurate endmember learning. This suggests that the correct endmembers are among the overestimated set. Nevertheless, since unmixing is an unsupervised process, it is not possible to directly identify the correct endmembers from the learned set. This limitation motivated the design of our proposed approach, where the number of endmembers is initially overestimated, and then the correct endmembers are automatically identified through the use of a clustering technique. Specifically, the CA clustering algorithm groups data instances into homogeneous clusters while simultaneously learning the optimal number of clusters, which corresponds to the number of endmembers in the image.

Several challenges are encountered in this module. First, the dataset designated for clustering is exceptionally small. Second, due to the nature of hyperspectral data, the dataset exhibits high dimensionality, with each data point being represented by a large number of bands, often exceeding 100 bands. Additionally, the clustering results are influenced by the initialization of the cluster centers and the chosen distance measure.

To address this, CA algorithm was performed with different initialization strategies. When the fuzzy membership matrix is randomly initialized, it consistently yields fewer endmembers than expected, with the average number of clusters ranging from one to two clusters. Alternatively, using Fuzzy-C-Means (FCM) to initialize the fuzzy membership matrix resulted in a slightly larger number of endmembers than the actual number. Finally, initializing the fuzzy membership matrix based on the correlation coefficient between the endmembers alleviates the randomness issue observed with random and FCM initialization methods. However, this approach generally leads to a larger number of endmembers compared to the other two methods. Although the correct number of endmembers was not always learned, the FCM-based initialization provided the closest approximation to the ground truth.

**Figure 7:**
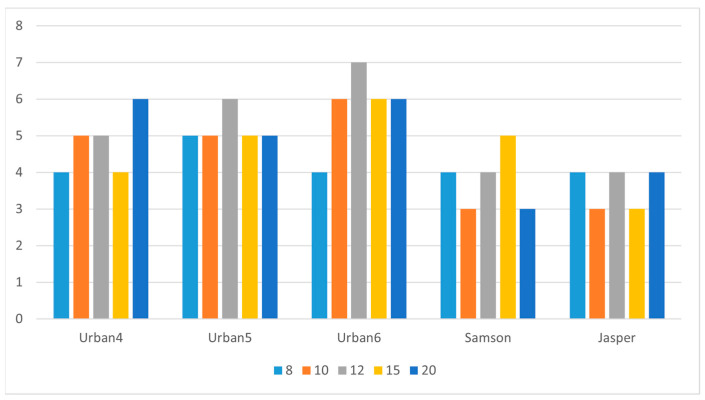
Number of endmembers learned when initially set as 8, 10, 12, 15, and 20.

Another factor affecting the clustering results is the distance measure. This effect is amplified by the high dimensionality of the data points. For this purpose, two distance metrics were explored: cosine distance and SAD. The experiments concluded that when SAD is employed, CA is able to learn the correct number of endmembers in most cases, and a close approximation in the remaining instances. Figure 7 reports the number of learned endmembers obtained after varying the initial number of endmembers (8, 10, 12, 15, and 20). As shown, the number of learned endmembers is very close to, or equal to, the ground truth number of endmembers. In fact, the proposed approach learned the correct number of endmembers in most cases, while deviating by just one unit (either +1 or −1) in a few.

## 5. Discussion

In this section, we discuss the results of the experiments where the number of endmembers was overestimated to 20. This value was chosen to be 3 to 5 times the actual number of endmembers for all datasets, ensuring a diversity of potential endmembers without significantly increasing the complexity or computational cost of the training. Accordingly, Table 8 presents the SAD scores for each pure material (endmember) present in the hyperspectral scenes of the considered datasets.

As can be observed, the Urban4 dataset was unmixed using six endmembers, deviating from the ground truth value of four. Notably, the Asphalt endmember exhibited a higher SAD compared to the Grass, Tree, and Roof endmembers. However, when a more refined labeling was used with five endmembers, the proposed approach successfully identified the correct number of endmembers. As a result, the SAD score for Asphalt decreased significantly, from 0.49 to 0.22.

**Figure 8:**
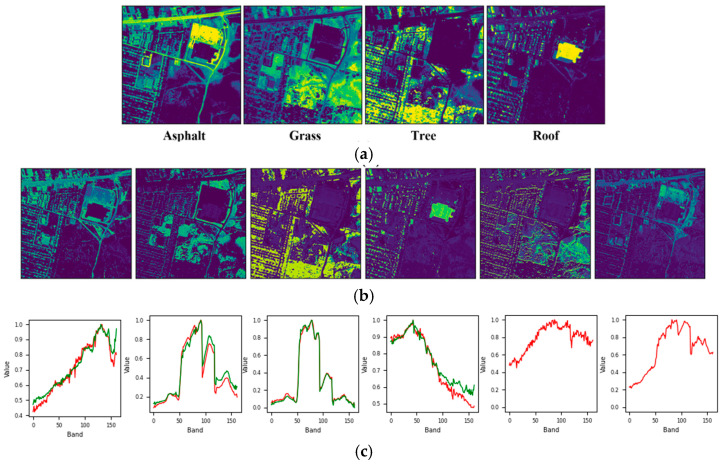
Unmixing results achieved by the proposed approach on the Urban4 dataset: (**a**) ground truth abundance maps, (**b**) learned abundance maps, and (**c**) ground truth endmembers (green) and learned endmembers (red).

**Figure 9:**
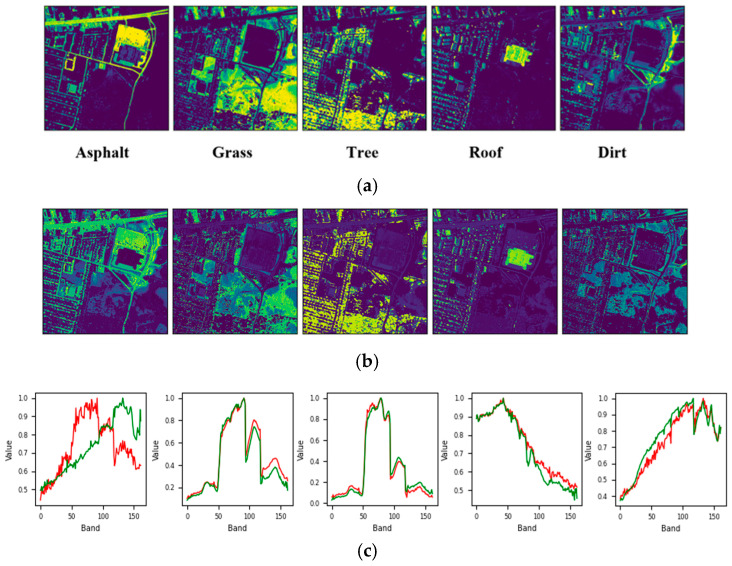
Unmixing results achieved by the proposed approach on the Urban5 dataset: (**a**) ground truth abundance maps, (**b**) learned abundance maps, and (**c**) ground truth endmembers (green) and learned endmembers (red).

**Figure 10:**
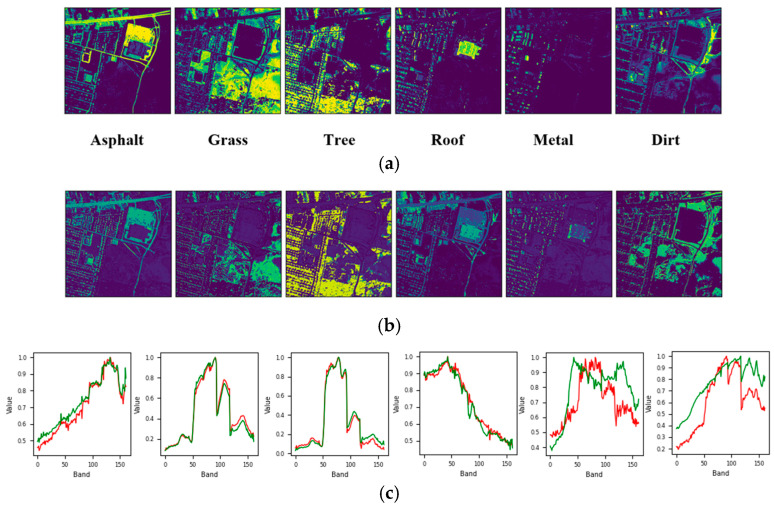
Unmixing results achieved by the proposed approach on the Urban6 dataset: (**a**) ground truth abundance maps, (**b**) learned abundance maps, and (**c**) ground truth endmembers (green) and learned endmembers (red).

Figure 8 and Figure 9 show that the reduction in SAD can be attributed to the initial mislabeling of Dirt as Asphalt, which simplified the dataset’s labeling but increased the difficulty of the unmixing process. Similarly, for the Urban6 dataset, which is labeled with six endmembers, the SAD score for Asphalt decreased further to 0.042. This outcome, along with the unmixing results shown in Figure 10, suggests that incorporating two additional labels (Dirt and Metal) contributed to a more effective unmixing process for Asphalt. However, it is important to note that these two materials exhibited relatively higher SAD values, approximately 0.185, likely due to their impure composition as mixtures of various materials.

**Figure 11:**
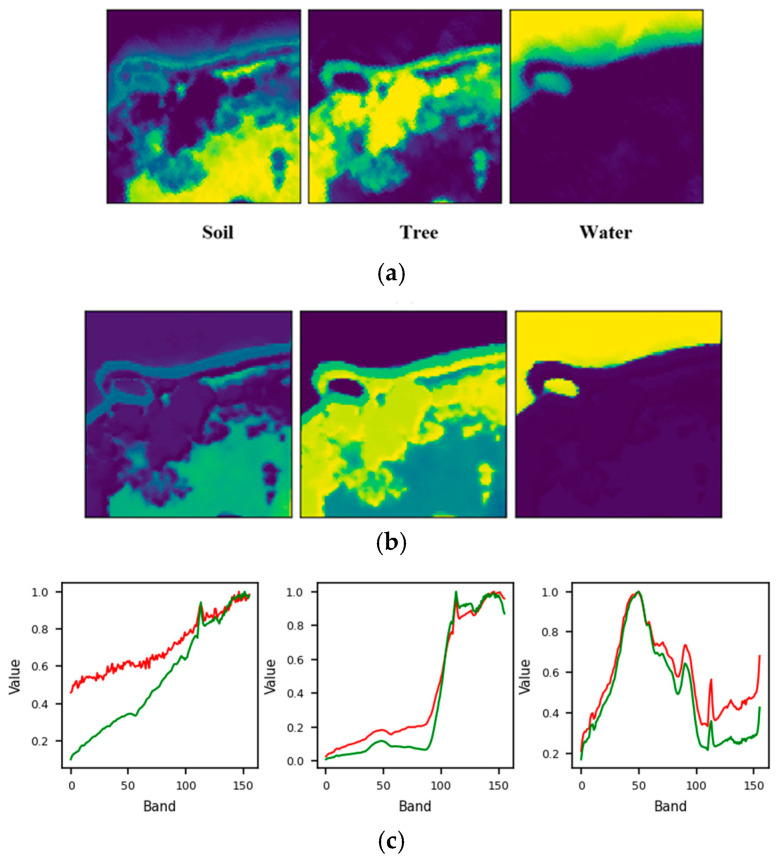
Unmixing results achieved by the proposed approach on the Samson dataset: (**a**) ground truth abundance maps, (**b**) learned abundance maps, and (**c**) ground truth endmembers (green) and learned endmembers (red).

**Figure 12:**
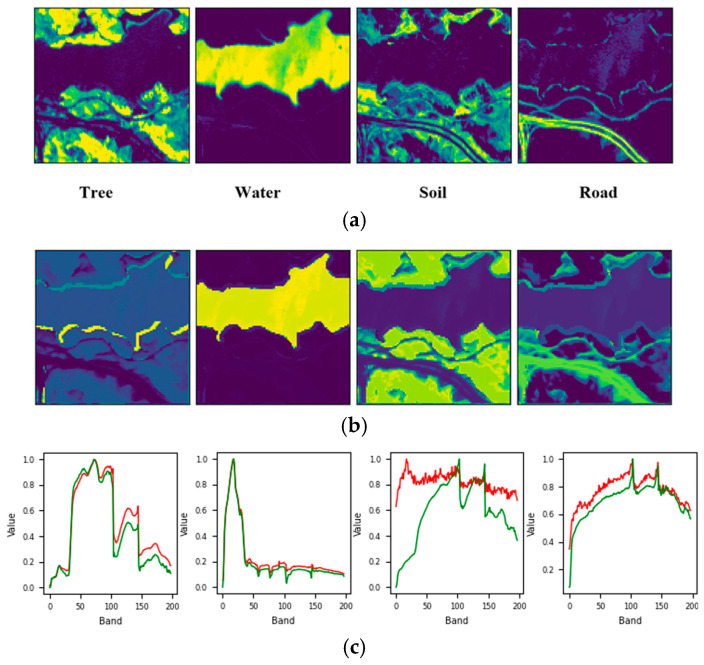
Unmixing results achieved by the proposed approach on the Jasper dataset: (**a**) ground truth abundance maps, (**b**) learned abundance maps, and (**c**) ground truth endmembers (green) and learned endmembers (red).

For the Samson dataset, the proposed approach accurately determined the endmember count, yielding comparable SAD scores of around 0.14 for Tree and Water, and a slightly higher SAD of 0.25 for Soil. The unmixing results depicted in Figure 11 further confirm this finding. On the other hand, the performance achieved using the Jasper data was also commendable, as the correct number of endmembers, equal to four, was successfully determined. Additionally, the SAD values for Water, Road, and Tree were low, while Soil exhibited a slightly higher SAD of 0.36. As seen in Figure 12, the soil fraction reveals instances where certain pixels show an exaggerated abundance. This observation implies that soil is intricately blended with another material.

Despite this complexity, our approach demonstrated commendable efficacy in distinguishing endmembers, even within complex datasets affected by noise and labeling inaccuracies. This highlights the robustness of our methodology across a range of diverse scenarios, showcasing its capacity to handle intricate situations and accurately identify the endmembers.

We evaluated the performance of our approach and compared it to various hyperspectral unmixing techniques from the three categories described in Section 2. The evaluation was carried out in regard to three aspects. First, the ability of the approach to determine the correct number of endmembers was assessed. Second, the accuracy of the identified endmembers was evaluated by comparing them to the ground truth signatures through the mean SAD (mSAD) [69], as introduced in Equation (3). SAD measures the angle between two spectra; thus, a lower SAD value indicates a higher similarity between the learned endmembers and the ground truth endmembers. Third, the learned abundances were compared to the ground truth abundance maps using RMSE [70], which is defined as

(14) $$RMSE=\sqrt{\frac{1}{R}\sum_{j=1}^{R}{\left\|{S_{j}-\hat{S}}_{j}\right\|}^{2}}\;$$

where ${\hat{S}}_{j}$ is the obtained abundance fraction with respect to endmember *j* and $S_{j}$ is the corresponding ground truth one. The lower the RMSE, the higher the resemblance between the obtained abundance maps and the reference abundance maps. The mean RMSE (mRMSE) is the average RMSE over all endmembers.

Table 9 reports the number of endmembers learned by six endmember estimation methods against the proposed approach. As can be seen, the proposed approach correctly estimated the number of endmembers for four out of the five datasets, significantly outperforming the other methods.

To evaluate the unmixing results, we first compared the performance of our approach against methods that only perform the unmixing while setting the number of endmembers as a constant (Category A) as discussed in Section 2.1. Then, we compared our approach to approaches that simultaneously estimate the number of endmembers along with the unmixing (Category B). This evaluation is in terms of the mSAD between the obtained endmembers and the ground truth endmembers. However, this comparison would only be fair with approaches from Category B, since Category A approaches have the advantage of knowing the number of endmembers while approaches from Category B do not. Nonetheless, as can be seen in Table 10, our approach outperforms all methods in Category B over all datasets. This implies that the learned endmembers resemble the reference endmembers more than the endmembers determined using the other three approaches do. In addition, it also outperforms the Category A methods with the Urban6 dataset, comes in second with the Urban5 and Jasper datasets, and ranks third with the Urban4 dataset.

Table 11 affirms the superior performance of our approach where it significantly outperforms methods from both categories except for the Samson and Jasper dataset in terms of mRMSE. Owing to the nature of the methods in Category A, it is to be expected to observe lower mRMSE since these methods have the number of endmembers known in advance. However, in the case of the Jasper dataset, our approach was outperformed by all approaches in Category B. Our higher mRMSE can be attributed to the fact that it estimated the correct number of endmembers while the other methods estimated more endmembers than are actually there in the image. Therefore, the mRMSE for the latter methods was averaged over more endmembers, lowering their total mRMSE.

## 6. Conclusions

This research sought to overcome the hyperspectral unmixing challenges through the design and implementation of a novel hyperspectral unmixing approach. Specifically, a CNN-based autoencoder was proposed to leverage both spatial and spectral information inherently available in hyperspectral images. The proposed approach employed a self-learning module specifically designed to determine the number of endmembers. This module relies on a fuzzy clustering algorithm to automatically learn the number of endmembers. Furthermore, a novel approach was devised to estimate the abundances of endmembers from the clustering outputs guided by the convex geometry model. The performance evaluation of the proposed approach showcased a significant performance improvement when compared to the methods that aim to simultaneously estimate the number of endmembers along with the unmixing. In particular, the proposed approach yielded SAD and RMSE enhancements of 47% and 42%, respectively. It also showed comparably excellent results when compared to hyperspectral unmixing methods that have prior knowledge about the number of endmembers. These results signify the potential for more accurate and reliable material identification in applications ranging from environmental monitoring to mineral exploration.

## Figures and Tables

**Table 1:** Characteristics of the three considered datasets.

Dataset	Image Size	Number of Endmembers	Number of Bands
Urban4	307 × 307	4	162
Urban5	307 × 307	5	162
Urban6	307 × 307	6	162
Samson	95 × 95	3	156
Jasper Ridge	100 × 100	4	198

**Table 2:** Autoencoder setting for unmixing hyperspectral data cubes.

Hyperparameter	Value
Patch size	80
Number of patches	150
Batch size	15
Learning rate	0.003
Epochs	250

**Table 3:** The average and minimum SAD of the obtained endmembers compared to the reference endmembers of the Urban4 dataset.

Number of Hidden Units	Average SAD (±std) of All Combinations	Minimum SAD of All Combinations
8	0.17776 ± 0.068887	0.051999
10	0.181732 ± 0.073206	0.040334
12	0.22191 ± 0.06381	0.06256
15	0.223169 ± 0.076408	0.041115
20	0.256954 ± 0.077475	0.053248

**Table 4:** The average and minimum SAD of the obtained endmembers compared to the reference endmembers of the Urban5 dataset.

Number of Hidden Units	Average SAD (±std) of All Combinations	Minimum SAD of All Combinations
8	0.148374 ± 0.050862	0.07297
10	0.169199 ± 0.056618	0.054721
12	0.184269 ± 0.055839	0.064277
15	0.198949 ± 0.056502	0.054085
20	0.216653 ± 0.057770	0.049290

**Table 5:** The average and minimum SAD of the obtained endmembers compared to the reference endmembers of the Urban6 dataset.

Number of Hidden Units	Average SAD (±std) of All Combinations	Minimum SAD of All Combinations
8	0.144711 ± 0.032459	0.088371
10	0.183636 ± 0.047738	0.092323
12	0.166801 ± 0.044830	0.082396
15	0.189501 ± 0.047678	0.074401
20	0.207844 ± 0.046994	0.073959

**Table 6:** The average and minimum SAD of the obtained endmembers compared to the reference endmembers of the Samson dataset.

Number of Hidden Units	Average SAD (±std) of All Combinations	Minimum SAD of All Combinations
6	0.204423 ± 0.128043	0.061324
8	0.227745 ± 0.119104	0.054205
10	0.234391 ± 0.122422	0.053254
12	0.277693 ± 0.114312	0.054807
15	0.281411 ± 0.109874	0.048858
20	0.322729 ± 0.105667	0.043728

**Table 7:** The average and minimum SAD of the obtained endmembers compared to the reference endmembers of the Jasper dataset.

Number of Hidden Units	Average SAD (±std) of All Combinations	Minimum SAD of All Combinations
8	0.265148 ± 0.106106	0.088756
10	0.306303 ± 0.106223	0.076265
12	0.279247 ± 0.090473	0.069970
15	0.273548 ± 0.092438	0.060158
20	0.313483 ± 0.092156	0.058683

**Table 8:** Average and minimum SAD for the obtained endmembers compared to the reference endmembers of the Jasper dataset.

Dataset	Actual Number of Endmembers	Obtained Number of Endmembers	Endmembers	SAD
Urban4	4	6	Asphalt	0.48848
			Grass	0.098442
			Tree	0.038957
			Roof	0.050862
Urban5	5	5	Asphalt	0.223371
			Grass	0.081140
			Tree	0.076126
			Roof	0.032651
			Dirt	0.063247
Urban6	6	6	Asphalt	0.042442
			Grass	0.043267
			Tree	0.080880
			Roof	0.031679
			Dirt	0.191048
			Metal	0.185489
Samson	3	3	Tree	0.134784
			Soil	0.255583
			Water	0.151655
Jasper	4	4	Water	0.077397
			Soil	0.359958
			Road	0.070194
			Tree	0.119996

**Table 9:** Comparison of the number of endmembers obtained by the proposed approach and other number-of-endmembers estimation methods.

Method	Urban4	Urban5	Urban6	Samson	Jasper
HFC [52]	37	37	37	8	7
HySime [15]	27	27	27	43	18
Agg. Clustering [56]	5	5	5	3	4
SPICE [60]	20	20	20	6	20
MASENE [63]	6	5	7	3	8
uDAS [21]	9	7	9	3	16
Our approach	6	5	6	3	4
GroundTruth	4	5	6	3	4

**Table 10:** Comparing the performance of the proposed approach against other methods in terms of mSAD. The best performances are shown in bold.

	Method	Urban4	Urban5	Urban6	Samson	Jasper
A	VCA [13]	0.411 ± 0.086	0.394 ± 0.056	0.214 ± 0.033	0.157 ± 0.132	0.186 ± 0.054
	CNNAEU [25]	**0.039 ± 0.003**	**0.075 ± 0.003**	0.122 ± 0.017	0.059 ± 0.030	0.224 ± 0.020
	Endnet [38]	0.050 ± 0.005	0.095 ± 0.008	0.157 ± 0.071	**0.024 ± 0.020**	**0.046 ± 0.050**
	SPICE [60]	0.627 ± 0.176	0.549 ± 0.117	0.567 ± 0.113	0.352 ± 0.183	0.530 ± 0.068
B	MASENE [63]	0.577 ± 0.209	0.446 ± 0.678	0.530 ± 0.113	0.209 ± 0.244	0.353 ± 0.113
	uDAS [21]	0.380 ± 0.128	0.243 ± 0.034	0.300 ± 0.077	0.324 ± 0.081	0.261 ± 0.081
	Our approach	0.164 ± 0.067	0.095 ± 0.074	**0.096 ± 0.073**	0.181 ± 0.065	0.157 ± 0.137

**Table 11:** Comparing the performance of the proposed approach against other methods in terms of mRMSE. The best performances are shown in bold.

	Method	Urban4	Urban5	Urban6	Samson	Jasper
A	VCA [13]	0.326 ± 0.007	0.330 ± 0.032	0.215 ± 0.004	0.316 ± 0.007	0.152 ± 0.005
	CNNAEU [25]	0.126 ± 0.002	0.167 ± 0.023	0.123 ± 0.004	**0.056 ± 0.011**	0.142 ± 0.001
	Endnet [38]	0.137 ± 0.0126	0.1313 ± 0.01	0.230 ± 0.081	0.102 ± 0.002	0.094 ± 0.006
	SPICE [60]	0.143 ± 0.003	0.127 ± 0.003	0.108 ± 0.003	0.266 ± 0.027	0.167 ± 0.028
B	MASENE [63]	0.149 ± 0.005	0.1446 ± 0.039	0.121 ± 0.001	0.186 ± 0.072	**0.082 ± 0.035**
	uDAS [21]	0.140 ± 0.008	0.131 ± 0.004	0.127 ± 0.013	0.241 ± 0.039	0.108 ± 0.034
	Our approach	**0.120 ± 0.024**	**0.073 ± 0.064**	**0.053 ± 0.032**	0.104 ± 0.075	0.218 ± 0.126

## Data Availability

The datasets in this study are openly available at https://rslab.ut.ac.ir/data. (accessed on 18 April 2025).
